# The contribution of media analysis to the evaluation of environmental interventions: the commuting and health in Cambridge study

**DOI:** 10.1186/1471-2458-14-482

**Published:** 2014-05-21

**Authors:** Joanna May Kesten, Simon Cohn, David Ogilvie

**Affiliations:** 1 MRC Epidemiology Unit and UKCRC Centre for Diet and Activity Research (CEDAR), University of Cambridge, Cambridge, UK; 2 Present address: School for Policy Studies, Faculty of Social Sciences and Law, University of Bristol, 8 Priory Road, Bristol BS8 1TZ, UK; 3 Institute of Public Health, Cambridge University, Forvie Site, Robinson Way, Cambridge CB2 0SR, UK

**Keywords:** Environmental interventions, Natural experiment, Media analysis, Print media, Qualitative research, Social media, Travel behaviour, UK

## Abstract

**Background:**

Media content can increase awareness of, and shape interactions with, public health interventions. As part of a natural experimental evaluation of the travel, physical activity and health impacts of the Cambridgeshire Guided Busway, we analysed print and social media discourse and interview data to understand the nature of new transport infrastructure and how it was experienced.

**Methods:**

Newspaper articles were systematically retrieved from the LexisNexis database and tweets were identified from an online archive. Interviews were conducted as part of the larger evaluation study with 38 adults. Inductive thematic analysis was performed and comparisons were drawn between datasets.

**Results:**

The findings are discussed in relation to five themes. First, an understanding of the intervention context and how the intervention was experienced was developed through accounts of events occurring pre and post the busway’s opening. Second, the media captured the dynamic nature of the intervention. Third, the media constructed idealised portrayals of the anticipated busway which in some cases were contradicted by the impact of the busway on the existing context and people’s lived experiences. Fourth, differential media coverage of the intervention components suggested that a lesser value was placed on promoting active travel compared with public transport. Lastly, interview data provided support for the hypothesis that the media increased awareness of the busway and served as a frame of reference for constructing expectations and comparing experiences.

**Conclusions:**

This analysis has contributed to the wider evaluation of the busway, helping to understand its nature and implementation and informing hypotheses about how the local population interact with the infrastructure by attending to the significance of representations in the media.

## Background

### Active travel, physical activity and health

Globally, physical inactivity is the fourth leading risk factor for mortality [1] and in the United Kingdom, the majority of adults do not meet physical activity recommendations [2]. Changes to the environment to encourage healthier travel choices are recognised as a potentially effective strategy for increasing population physical activity [2-4]. Active travel, in particular active commuting, is associated with higher total physical activity [5,6] and physical wellbeing [7] and lower cardiovascular risk [8,9]. Over half of journeys in the UK are made by car [10], modelling suggests that increases in active travel and reductions in motor vehicle use would have public health benefits [11], and promoting the use of public transport can facilitate walking [12,13] and cycling [14].

### Natural experimental studies in public health

Ecological models in health research acknowledge the interdependence between individuals, their environment and their health [15,16]. Health promotion efforts directed at high-risk individuals may be less effective in reducing disease prevalence than efforts to shift the entire population distribution of a risk factor [17]. Health-enhancing modifications to the environment may have the capacity to reach large ‘exposed’ populations [18,19]. However, randomised controlled trials are often not feasible for assessing environmental interventions and the evaluation of ‘natural experiments’, in which the allocation of interventions cannot be manipulated by the researcher, presents a number of challenges. These include defining comparison groups, minimising differences between comparison and intervention groups at baseline in the absence of randomisation, determining the level of exposure to the intervention and attributing outcomes to the intervention [20,21]. Novel approaches to understanding the context and mechanisms operating within natural experimental studies may therefore help in the elucidation of causal understanding. Such approaches are in line with the realist evaluation configuration ‘Context-Mechanism-Outcome’ - the hypothesis that intervention outcomes are brought about through context-specific mechanisms [22].

### The Cambridgeshire guided busway

The Cambridgeshire Guided Busway (hereafter the ‘busway’) is a modification to the physical environment that supports active travel and public transport. The busway was introduced to address increasing congestion on major roads and ‘rat running’ through small villages associated with car commuting into Cambridge, UK [23]. The busway is a piece of transport infrastructure, connecting St Ives, Cambridge and Trumpington, which consists of a guideway for buses and a ‘maintenance track’ for emergency vehicles, pedestrians, cyclists and horse riders. Guided bus technology ensures continuous contact between the bus and the kerb of the track and allows the buses to use normal roads as well as the guideway.

The Commuting and Health in Cambridge study is a natural experimental study designed to assess the impact of the busway on travel behaviour, physical activity and health. The study protocol has been published in detail elsewhere [24] and describes a quasi-experimental cohort study of adult commuters, including nested in-depth quantitative and qualitative components. To be eligible for the study participants had to be over 16 years of age and travel to work in Cambridge from within a radius of approximately 30 km. Four annual waves (2009 to 2012) of postal questionnaires and (optional) objective physical activity measurement were conducted before, during and after the opening of the busway in 2011. A complementary intercept survey of busway users was performed in 2012 to assess who used the busway, for what purposes and how such journeys would have been made prior to the busway. Qualitative fieldwork was conducted in each year of the study to gain insights into the views and experiences of participants. Previous qualitative and mixed-method papers from the study have examined the social context of commuting practices [25], the socioeconomic structure of car commuting [26], depictions of wellbeing associated with commuting [25], the resilience of active commuters to apparently hostile commuting environments [27], factors underlying changes in commuting practices following home or work relocation [28] and the initial experiences of busway users [29]. The various components of the study combine to provide novel contributions to the understanding of the links between environmental change and travel and physical activity behaviour change. The current analysis provides an additional lens through which to examine the interaction between the busway and its context.

### Discourse of the media

**Figure 1:**
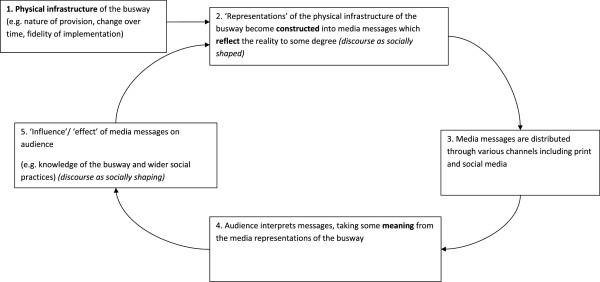
Model of the relationship between the media and the busway.

In evaluating interventions, realist theorists propose that it can be helpful to understand the contexts in which an intervention works or does not work [22], in particular how components of the intervention interact with each other and their context [21,30]. Discourse (the use of language) is a social practice in that it is both ‘socially shaped’ and ‘socially shaping’ [31]. It has a central place in modern society, is receptive to social change [31], and can therefore act as both a component of and a contributor to the context of an intervention such as the busway which is experienced through social practices. To some extent, in its capacity as a wide-reaching information source, the media can define the terms in which we think about the world by both reflecting and constructing reality [31-33]. We therefore postulated that media representations mediate how the busway is understood and experienced and become part of the intervention [32,33]. We developed a model of the relationship between the media and the busway (Figure 1) informed by Fairclough’s Critical Discourse Analysis and Hall’s work on the media [31,32]. The tangible issue of the busway must first be transformed into media discourse that represents the issue. In constructing media discourse there is the potential to modify what is understood of the busway [34]. Audiences process and take some meaning from distributed discourse [34]. The interpretation and response may vary depending on the audience [31] and can be difficult to measure [32]. Responses to both the media and the intervention may feed back into media discourse.

### Media analysis in the commuting and health in Cambridge study

Unlike some components of the Commuting and Health in Cambridge study, a media analysis is able to capture the complete timeline of the busway’s history. Whilst a previous ethnographic study focused on the ‘micro-level’ experiences of individuals and small groups of people who found themselves adopting and adapting to the busway in the first weeks after its opening [29], a media analysis can also address the ‘macro-level’ discourses surrounding the busway over the longer term, for example, exploring the opinions of stakeholders such as those in local government, and can allow the interview findings from the different phases of the study to be placed within the social context of the media’s representations. More specifically, analysis of media discourse, provides an opportunity to examine potential mechanisms behind the observation that some members of the study cohort reported walking and cycling to work despite an apparently unsupportive environment [27]. The authors of that analysis suggested that participants may have been representing a general public discourse around road safety rather than their own experience. This warrants a further exploration of how public discourses are constructed through media representations.

Most media analyses in public health research have adopted a variety of quantitative methods, drawing inferences about the impact of media discourse by quantifying the themes covered [35-37] or assessing the longitudinal associations between media coverage and the incidence of behaviours, e.g. smoking cessation [38-40]. Others have conducted manifest content analyses using grounded theory to generate themes and have then quantified the number of related media stories [41-44]. Fewer studies have taken an in-depth, inductive approach to understanding the role of the media in public health issues [45,46], which may enable the exploration of meaning and mechanisms [21].

This multi-method paper, aligned with the realist evaluation approach [22], examines the discourse of the media to understand the nature, context, implementation and experience of an environmental intervention —the busway — and the consequences of media consumption on experiences of the intervention. To investigate the latter question further, we supplement media data with themes identified in interviews conducted with local residents as part of the Commuting and Health in Cambridge study. By using more than one data source we aim to generate a deeper and more complete understanding of the media’s contribution to the evaluation and understanding of environmental interventions [47].

## Methods

### Data sources

There are various relevant traditional and social media with which we could have engaged including magazines, newspapers, websites, several forms of social media (such as Twitter and Facebook) and television. This analysis utilised newspaper and Twitter data sources. Newspapers provide news reporting which reaches a high proportion of the population [48]. The relationship between print and social media has been described as cyclic, whereby print media are both driven by and a driver of social media [49]. Twitter is a micro-blogging service allowing networks of people ‘*to communicate and stay connected through the exchange of quick, frequent messages’*[50]. Twitter facilitates conversation within a ‘shared social context’, unconstrained by geographical setting and the timing of response [51]. The real-time nature of tweets means that Twitter is a useful tool by which to spread ‘breaking news’ [51].

### Data collection

The LexisNexis database was searched for UK newspaper articles dating from October 21, 2004 (when the busway was first featured) to November 21, 2012, spanning coverage before and after the opening of the busway on August 7, 2011. LexisNexis archives 703 UK newspapers including broadsheet, tabloid and local titles [52]. Local newspapers serving the area of the busway include the Cambridge Evening News; Cambridge First; Ely Standard; Fenland Citizen; Hunts Post; and Cambs Times.

The following search strategy was developed from an initial scoping of keywords in media discourse:

"Guided bus OR busway OR guideway OR misguided [a pun used to describe the busway] OR bus! [exclamation mark searches for all variations of the root term]* OR buses OR guided OR park OR ride AND Cambridge!* OR Cambridgeshire OR Huntingdon! OR St Ives OR Trumpington OR Longstanton OR Histon OR Addenbrookes OR Peterborough."

The retrieved articles were then filtered by the database to identify articles including the term ‘busway’. The approach of this search is, therefore, systematic but not exhaustive.

An online database ( http://www.topsy.com) was used to search tweets retrospectively. This database is limited by an inconsistent searching capacity and the earliest stored tweets are from 2008. For this reason the search was continued until theoretical saturation of key concepts had been achieved, i.e. little new information was emerging [53]. The Twitter keywords were similar to those of the newspaper search, but owing to the unsophisticated search engine available it was necessary to run multiple searches with short combinations of terms (e.g. "Guided Bus Cambridge").

Interview participants were purposively sampled from adults who had taken part in the Commuting and Health in Cambridge study, either as part of the main cohort who had completed annual data collection over a maximum of four years or by completing the intercept survey. The intercept survey participants represented more diverse social positions than the main cohort, thereby providing the opportunity to sample from a broader cross-section of social groups. Participants representing a range of characteristics (gender; age; education; and home location, used as an indicator of exposure to the busway) were invited to participate by letter. Once informed consent had been obtained, semi-structured interviews were conducted between February and June 2013. Interviews were conducted until theoretical saturation was reached [53]. Interviews were performed in batches and continued until a broad range of participants had been interviewed. The Cambridge Psychology Research Ethics Committee Ethical granted approval for this study (Ethics reference number Pre.2012.14). Ethical approval was not required for the analysis of media discourse which was already in the public domain. The interviews explored commuting experiences; facilitators, barriers and the process of travel behaviour change; and the perceived impact of the busway on these behaviours. The interview topic guide did not explicitly mention the media, although participants were asked about their decision to use the busway if they had done so; the media were raised spontaneously by 12 of the 38 interview participants when discussing perceptions of the busway.

### Analysis

Media articles and tweets were included for analysis if the inclusion and exclusion criteria were satisfied (Table 1).

**Table 1:** Inclusion criteria for the newspaper and twitter search

**Inclusion criteria**	**Exclusion criteria**
Busway was the primary topic of more than 50% of the content	Duplicate articles or tweets
Term ‘busway’ included in newspaper article or title (this criterion was not used for the Twitter search)	Coverage of other busways not in Cambridgeshire

In-depth qualitative analysis of all data sources involved systematic inductive coding facilitated by QSR NVivo 8 [54]. An iterative process was used to identify salient themes — defined as those that were relevant, repeated and meaningful — from these initial codes and to draw comparisons between data sources [54]. Within the interview data, we inductively coded and extracted themes relating to the media coverage of the busway. JK conducted the interviews and coding, the latter being refined in collaboration with DO and SC. JK and DO had used the busway (including the maintenance track) and were familiar with many of the themes emergent within the media analysis and interviews. This familiarity and prior knowledge helped inform the research question, data collection and interpretation. During the interviews JK did not disclose her experiences or views of the busway in an attempt to remain neutral and minimise participant response bias (provision of responses which the participant believes the interviewer would like). Following analysis, quotes reflecting each theme including both dominant and divergent cases were selected to illustrate the findings. Whilst the analysis was broadly inductive in nature, it was inevitably informed to some extent by the aim of understanding the complex nature, context, implementation and experience of an environmental intervention – components of the realist evaluation approach [22]. This study adheres to the RATS guidelines for reporting qualitative research [54].

## Results and discussion

**Figure 2:**
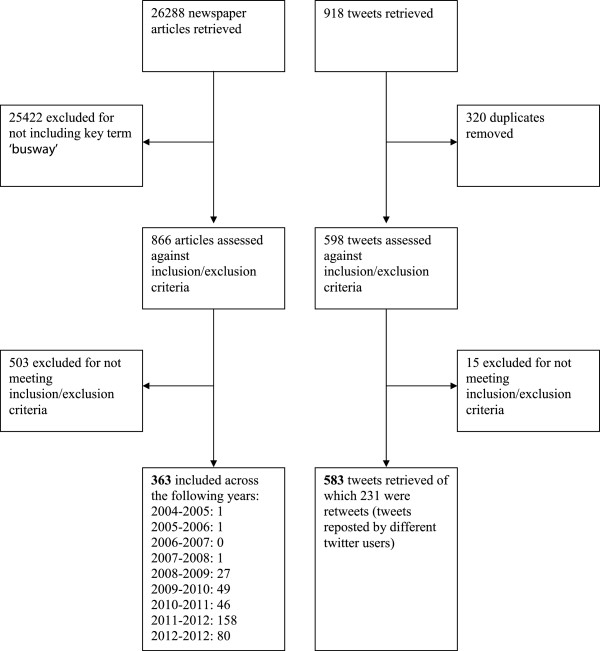
Flow diagram of included newspaper articles and tweets.

Three hundred and sixty three newspaper articles and five hundred and eighty three tweets met the inclusion criteria (Figure 2).

The characteristics of the 38 interview participants are presented in Table 2. In total 132 participants were invited to participate in an interview (32 cohort members and 100 intercept survey participants), of whom 38 (29%) consented to an interview. The response rate was higher among cohort members (72%) than among intercept survey participants (15%).

**Table 2:** Interview participant characteristics (n = 38)

**Characteristic**	**Subcategory**	**N**
Gender		
	Male	17
	Female	21
Age (years)		
	30 – 39	7
	40 – 49	6
	50 – 59	16
	60 – 69	7
	70 and over	2
Employment		
	Employed	35
	Unemployed^1^	3
Education		
	Higher education (postgraduate qualification, degree, NVQ4, NVQ5 or equivalent)	19
	Secondary education (A-level, GCSE, BTEC, GCE)	12
	Other qualification	4
	None	3
Recruitment group		
	Intercept	15
	Cohort	23
Travel behaviour change^2^		
	No change	11
	More active or decreased car use	12
	Less active or increased car use	11
	Change which does not affect activity levels or car use	4
Interviewed previously		
	Yes	3
	No	35
Total		38

^1^ Includes: retired or looking after home or family.

^2^ Behaviour change self-reported during interviews.

The five themes which emerged from the thematic analysis are developed below and summarised in Table 3.

**Table 3:** Summary of key findings

**Theme and description**	
Context of the busway	
•	Accounts of events occurring pre and post the busway’s opening developed an understanding of the intervention context and how the intervention was experienced.
•	For example, contradictory attitudes towards the busway amongst the key stakeholders developed a context of limited acceptability of and perceived need for the busway.
Dynamic nature of the busway	
•	The media captured the dynamic nature of the intervention including its phased completion and capacity to adapt to varying demands over time.
•	For instance, the implementation of the busway could not be represented by a clear dichotomy of ‘available’ vs. ‘unavailable’.
Idealised and lived experiences	
•	The media constructed idealised portrayals of the anticipated busway in terms of its reliability, frequency, speed and value for money, which in some cases were contradicted by the impact of the busway on the existing context and people’s lived experiences.
Prioritisation of the busway over the maintenance track	
•	Differential media coverage of the intervention components suggested that a lesser value was placed on promoting active travel compared with public transport.
•	This unequal distribution of discourse both reflected and contributed to the lesser priority attached by stakeholders to the maintenance track compared with the busway itself.
•	The name ‘maintenance track’ does not emphasise the opportunity for active travel.
Consumption of media content	
•	Interview data supported the hypothesis that the media increased awareness of the busway and served as a frame of reference for constructing expectations and comparing experiences.
•	For those who had not used the busway, the media coverage offered an indication of how it was experienced and influenced willingness to try the busway.

### Context of the busway

The discourse of the media facilitated an understanding of the social system into which the busway was introduced in the periods before and after the opening of the busway.

#### Pre-opening

Media coverage revealed tensions between contradictory attitudes towards the busway amongst the key stakeholders, namely the public, the local authority funding the intervention (Cambridgeshire County Council), the contractor (BAM Nuttall), local campaign groups (such as CastIron and the Cambridge Cycling Campaign) and various political parties. The public consultation on the proposal to build the busway primarily featured objections from campaign groups who wanted a disused railway (on which the busway was to be constructed) to be reopened, and from those who viewed the current bus services as sufficient. These objections developed a context of limited acceptability of and perceived need for the busway, both of which may be important for the effective implementation of new transport systems [55].

*"People don’t want to catch buses because, unlike trains, they are seen as cheap and demeaning. Outside London, you tend to see only women with children, pensioners and students catching buses."* The Times, October 7, 2005

Counterarguments from stakeholders indicated the anticipated benefits of the busway.

*"This is a major investment by Stagecoach in a project that we believe will bring significant economic, transport and environmental benefits to the Cambridgeshire region."* The Scotsman, October 7, 2008

Before these anticipated benefits could be realised, complications with construction caused a two-year delay in completion and a large overspend which were reiterated in the media. The construction problems were the subject of reported conflicts between the local authority and the contractor who disputed liability for the overspend.

*"£55 million guided bus court battle launched: Council bosses in Cambridgeshire have launched a n…* *http://t.co/8syuZJl* *#Cambridgeshire"* Tweet: Cambridgeshire News, September 2, 2011

Through these disputes, the busway became a politicised matter in which the public were depicted as being detrimentally affected.

*"The Tory administration is allowing the guided bus contract to bleed taxpayers’ money while it buries its head in the sand…"* Cambridge Evening News, June 21, 2010

#### Post-opening

Once the busway had opened, positive and negative effects on the local context were noted. To facilitate reliability and speed, for example, buses on the busway were given priority at traffic lights; this was negatively received by motorists. On the other hand, the media also depicted unexpected economic benefits of the busway.

*"Businesses in the market towns of St Ives and Huntingdon have also been boosted by the number of people travelling out from Cambridge."* Cambridge Evening News, August 15, 2011

These aspects of the media discourse support a more general observation that when interventions of this kind are introduced to a dynamic system, it can be difficult to determine the extent to which outcomes may be attributable to the intervention above any naturally occurring changes in the system [22].

### Dynamic nature of the busway

Media coverage depicted the dynamic nature of the busway in terms of its phased completion and capacity to adapt to varying demands over time. It is apparent, therefore, that the busway was not an inert modification to the environment, but a dynamically changing intervention [56].

With a highly publicised intervention such as the busway, the media provide a means of exploring dynamic elements and "tipping points" — a term used to describe events preceding a change that has some impact on the outcome [57]. For instance, initial popularity of the system led to overcrowded buses which affected the reliability of the service. The bus operators responded by adding more buses and modifying routes and timetables to improve reliability.

*"The busway is busy but to the detriment of the passengers."* Cambridge Evening News, January 11, 2012

*"This new [bus] stop will help encourage even more services for the town and make it easier to use the busway, as well as reducing bus times."* Cambridge Evening News, January 11, 2012

This extract illustrates how *spatial* exposure to the busway was altered by the introduction of a bus stop. Similarly, sections of the maintenance track were reportedly vulnerable to flooding, which further illustrates the potential for effective exposure and access to the intervention to vary in time and space.

Assessing the demand for services took time and therefore the adaptations to initial overcrowding — which could be described as a tipping point — were not immediate. Possible outcomes from this tipping point included: no change in the number of people using the busway, because new buses were expected to *"help cope with the demand"*; more people using the busway, because of the increased capacity; or fewer people using the busway, because the initial experience of overcrowding acted as a barrier to further use. There was little in the media content to support the latter hypothesis, although reported negative experiences could precede such an outcome. Alternatively, the lack of evidence could mean that people were generally tolerant of the adjustments. These hypothetical outcomes derived from the media analysis can be transformed into hypotheses to be tested empirically, in much the same way as hypothetical ‘virtuous spiral’ and ‘vicious spiral’ vignettes have been used to guide the evaluation of the health impacts of urban motorway construction in Glasgow [58].

The *timing* of exposure to the maintenance track is difficult to determine because walkers and cyclists reportedly used the track before construction finished.

*"Cambridgeshire’s guided bus track is proving a hit even before it is up and running with cyclists. Bike enthusiasts have been taking to the smooth concrete roadway to explore the countryside near their homes, and some have even been using it as a cycleway to get to and from work."* Cambridge Evening News, May 30, 2009

Busway officials also stressed *"that commuter numbers would shoot up after the summer holidays"* (Cambridge First, August 18, 2011), referring to expected student passengers. It was clear that the implementation of the busway could not be represented by a clear dichotomy of ‘available’ vs. ‘unavailable’ and that sustained changes in behaviour might take time to develop.

Capturing tipping points and the dynamic elements of the busway, such as changing levels of exposure to the intervention, and testing their association with the outcome of interest poses challenges for evaluation, particularly when the implementation of ‘natural experimental’ interventions is outside the researcher’s control [20]. For this reason, it is desirable that the intervention fidelity and time-varying exposures should be monitored [59] and, where appropriate, incorporated into statistical analyses. However, this can be difficult if the relevant events in the natural history of an intervention are unpredictable, unobserved or imperfectly measured [20]. It may sometimes be more feasible to use the knowledge of implementation mechanisms, gained from data sources such as the media and qualitative interviews, to inform the interpretation of quantitative analysis and the modelling of the potential impact of directly observed changes using methods such as systems dynamic modelling [60,61].

In summary, the discourse of the media in relation to the context and dynamic nature of the busway highlight the potential complexity of evaluating an intervention that involves multiple interacting social and physical components [62] which have been introduced in stages.

### Idealised and lived experiences

In addition to capturing how the intervention was implemented, the media constructed idealised portrayals of the anticipated busway which in some cases were confirmed or contradicted in all three sources of data by the impact of the busway on the existing context and the reported lived experiences [32].

To encourage patronage, promotional media discourses from stakeholders emphasised anticipated benefits of the busway, such as that it would provide reliable, frequent, fast, good value for money, all of which are known to be important in the selection of mode of transport for commuting [28], as well as those relating to a smooth, comfortable ride and scenic views. The latter relates to previous research in this study suggesting that journeys to work can have affirmative implications for wellbeing [25].

*"We think [people] will be attracted by the smoothness of the ride, leather seats and free Wi-Fi. We want people to be able to ride on the busway having a cup of coffee with their laptop open, catching up on e-mails."* The Times, January 26, 2009

Some of these busway features were also commented on by Twitter users, affirming that they were positively experienced as ‘anticipated’ and signifying that the print media were contributing to social media to some extent.

The emphasis placed on comfort features may reflect an intention to encourage people who would not normally use buses to shift to the busway.

*"We are looking to appeal to people who are normally using their BMW to go into Cambridge."* Cambridge Evening News, April 17, 2009

In practice, some of the positive features of the busway did not deliver as advertised. Such opposing accounts were subsequently reincorporated and reflected by the media discourse. This process supports the hypothesis of the model presented in Figure 1 that audience response to both the media and the intervention feed back into media discourse.

Comparisons between the idealised and realised experiences of the busway can be related to the concept of intervention fidelity. For example, the use of normal roads for part of the route meant that busway travel was not as fast as anticipated.

*"—‘s letter represents the beginning of the public’s realisation that the guided busway does not improve journey times or reliability for the vast majority of typical journeys. The explanation is simple and two-fold: Two-thirds of the timetabled journey is on ordinary roads, mixed in with the traffic just the same as before the guideway was built."* Hunts Post, January 4, 2012

Lack of intervention fidelity, as illustrated above, has previously been highlighted as an explanation for non-significant changes in walking after relocation to ‘livable neighbourhoods’ [59].

Negative comparisons were also made between the speed of the busway and the pre-existing public transport system.

*"St Ives bus station to Cambridge bus station, 2002: 35 minutes. By £181 million Guided Bus from Aug 2011: 36 mins."* Tweet: Chris Rand, June 6, 2011

The busway offered more bus stops than some previous public transport routes. While this may have made the new service more accessible, it was also reportedly slower as a result.

*"The 55 was quicker. The one thing I would say about this service is that it has opened up more stops and so takes longer for me to get home. But I intend to carry on using this service. Nothing is worth parking in Cambridge."* Hunts Post, August 17, 2011

Although this passenger was unhappy with the service, he acknowledged that it was still the most viable option and continued to use it. Previous mixed-methods research in this study has examined potential explanations for the reporting of walking and cycling to work despite perceived unsupportive environments [27]. Guell and colleagues suggest that people may have developed strategies to cope with these unsupportive conditions; may have been representing a general public discourse rather than their own experience; and may have reasons for relying on active commuting despite adverse environments, such as employers’ restrictions on car parking. The latter explanation illustrates the importance of highlighting not only the nature of the intervention but also perceptions of its context including the available alternatives. As Pawson and Tilley have suggested, interventions "work, if subjects choose to make them work and are placed in the right conditions to enable them to do so" [63] (p294).

Initial impressions of the busway appeared important to users, and because the local authority had asserted in the print media that individual benefits would be seen immediately (*"guaranteed journey time",* Cambridge Evening News, December 17, 2010) when these were not realised, some users reported being reluctant to continue using the service.

*"…If it can’t run on time on its first working week day, may stick with the car."* Hunts Post, August 10, 2011

Initial experiences of the busway varied depending on the transport mode previously used. Car users tended to experience the busway as positive and novel, whilst previous bus users had more mixed impressions.

*"To my surprise the 7.58 am Stagecoach service arrived ahead of the 7.48 am, baffling new commuters to the rapid transport system. Onboard most passengers were distracted by the morning’s hazy sunshine over Fen Drayton Lakes - a welcome relief to the lorries on the A14."* Cambridge First, August 11, 2011

Although the user experience was varied, this analysis supports previous research in this study [29] in suggesting that motorists tended to experience the busway more positively than previous public transport users. This suggests the possibility of differential effects on behavioural outcomes depending on baseline travel behaviours, which should be taken into account in quantitative longitudinal analyses [64].

References made to other new busway users imply an element of collective experience. Previous ethnographic research on the busway found evidence of passengers new to the busway collectively learning about how to use the system [29]. Collective experiences were both positive and negative:

*"…On the first day everyone was beaming which was nice to see."* Cambridge First, August 11, 2011

*"RT @crispincooper: Doesn’t everyone just LOVE the mis-guided bus scheme?* *http://bit.ly/f80TK6* *#cambridge @julianhuppert"* Tweet: SITP Cambridge, December 2, 2010

The latter tweet also displays, using conversational discourse, how Twitter acted as a platform for public debate on the merits of the busway. The Twitter data also suggested that the public were becoming over-exposed to the print busway coverage:

*"We’re bored of your pointless busway stories now "@CambridgeNewsUK: Guided bus breaks down amid Cambridge’s rush hour* *http://t.co/M6Tnwr4g* *""* Tweet: Emma, November 1, 2011

By re-tweeting this print article and using ‘we’re’ to personify the Twitter community and wider public, this user reconceptualised the print media discourse as being uninteresting.

### Prioritisation of the busway over the maintenance track

The print media, in particular, featured the busway more than the maintenance track which provides access for emergency vehicles and serves as a route for pedestrians and cyclists. Unequal distribution of media discourse both reflected and contributed to the lesser priority attached by stakeholders to the maintenance track compared with the busway itself. For instance, early reports of busway usage did not include the maintenance track, suggesting that it may have been of less importance to the local authority.

*"Almost 56,000 trips made on the Cambridge #busway in the first 7 days. I wonder how many cyclists use the path."* Tweet: Cambridge Cycling Campaign, August 16, 2011

The name ‘maintenance track’ was commented on by the Cambridge Cycling Campaign because it did not emphasise the opportunity for active travel.

*"The cycleway next to the guided busway isn’t a great name and technically, ‘The Busway’, refers to the bit the bus is meant to use, not the service road alongside, which is also for the use of cyclists and pedestrians."* Cambridge Evening News, June 6, 2011

The absence of lighting along the maintenance track was criticised as dangerous and a barrier to its use.

*"Cyclists have been injured in accidents caused by a lack of lighting on the track next to the guided busway, it is claimed. Riders, particularly women and the elderly, say they fear for their safety on dark stretches of the route and a petition has been launched calling for the county council to take urgent action before anyone is seriously hurt."* Cambridge Evening News, February 11, 2012

The emphasis in the above extract on certain demographic groups — such as women and older adults, who are already less well represented amongst cyclists and may be particularly deterred by the lack of lighting on the maintenance track — could further perpetuate the perception that cycling is unsafe amongst these groups and therefore act as a barrier to cycling.

The local authority’s reported response to the lack of lighting along the maintenance track did not appear to acknowledge the importance of an environment perceived to be supportive of walking and cycling [65,66].

*"Lighting on the busway was limited to junctions and stops to minimise the impact of the busway on the local environment and ecology."* Cambridge Evening News, February 11, 2012

Despite the maintenance track having received less media coverage and some criticism, some positive features were also presented in the media discourse. For example, the transport charity Sustrans allocated some funding to add a smooth surface to the maintenance track.

In addition, the maintenance track was perceived to offer an alternative to the busway itself and was positively experienced by some users. These users may have been resilient to less supportive environments, perhaps because they were experienced cyclists.

*"I could almost be in the Netherlands…lovely wide cycle path alongside the Cambridgeshire guided busway."* Tweet: Harry Rutter, December 11, 2012

By reporting these positive experiences, the discourse of the media (predominantly Twitter) counteracted the unequal coverage in the print media to some extent.

In summary, the opportunity to promote use of the maintenance track through the media could have been capitalised upon more.

### Consumption of media content

The model in Figure 1 hypothesises that the media can mediate how the intervention is understood and experienced by using representations. Using the interview data to complement the media sources offered the opportunity to explore the consumption of the media [34] and whether this hypothesis could be supported.

When discussing views of the busway, themes relating to the media were raised spontaneously by interview participants, suggesting that the media informed and helped construct expectations of the busway. Indeed, awareness of the busway and its narrative was attained in part from the media.

*"We heard about [the busway] on the news, that was the first thing on the local news and through the papers"* (Woman, 60–69 years)

Similarly, the reverberation of phraseology from the media coverage suggested that it served as a frame of reference to construct expectations and against which to compare experiences, but also that the media representations could not be disentangled from the busway itself – in other words, that talk about the busway and about its media representations were synonymous. For those who had not used the busway, the media coverage offered an indication of how it was experienced and influenced willingness to try the busway.

*"I heard all the original stories about the mis-guided bus and all the other stuff and all the problems. But I’ve only heard good reports since, the people who use it seem pretty happy with it and I even said to my other half, "I really must take a trip to St. Ives sometime on the Guided Bus to see what it’s all about""* (Woman, 60–69 years)

The use of the terminology ‘mis-guided bus’ points to some absorption of media discourse into public discourse.

Elements of the media discourse were disputed by some users of the busway, illustrating that the media were used as a frame of reference with which to compare experiences. Thus the media discourse could be challenged by lived experiences.

*"The busway which is not the comfortable thing [the stakeholders portrayed in the media] said it was going to be. You can go down it with your cup of coffee and nothing will move but the buses do move, every time you go over one of the connecting bits there’s a jump, jolt so it’s not as quiet and smooth as it was said to be."* (Man, 60–69 years)

This extract disputes the aforementioned idealised discourse of the media.

Finally, negative media discourse relating to the busway and public transport was perceived to discourage its use.

*"All you hear is the buses are late all the time, that the drivers are awful, everything you read in the papers about buses is bad."* (Man, 50–59 years)

In summary, whilst media coverage may mediate expectations and influence people’s willingness to trial a new intervention, it does not override the importance of lived experiences.

## Conclusions

The examination of media narratives has allowed us to understand more about the nature of the intervention and the context in which it was implemented. Comparisons between more than one data source produced a rich and novel dataset, and the use of qualitative methods to interpret the data has produced an in-depth insight into the discourse of the media representing the busway. However, it is important to acknowledge that media discourse can be biased (for example by deliberately exaggerating or polarising issues) in pursuit of an aim to achieve high readership levels with concise reporting [33], and that our analysis did not encompass data representative of all media, e.g. television or other social media channels. In addition, the interviews were not originally designed to assess the impact of the media on perceptions of the busway, and may therefore not have elicited all relevant insights from participants in this regard. The interview sample included a higher proportion of cohort members than intercept participants and within the main cohort, a large proportion had been educated to degree level, although the recruitment of intercept participants to some extent off-sets this. The interviewed sample did not represent the experiences of younger adults (less than 30 years) and included a high proportion of older adults.

Exploring the media’s discourse provided insights into the nature and experience of the busway, eliciting themes relating to the context of the busway, its dynamic nature, the contrasts between idealised and lived experiences, and the different priorities applied to different elements of the infrastructure. Media analysis provides a way of capturing and understanding the dynamic and complex elements of an environmental intervention in a natural experimental study. This analysis has contributed to the wider evaluation of the busway intervention, helping to understand the nature of the intervention and how it was implemented. For example, it has clarified the potential for exposure and access to the intervention and the fidelity of its implementation to vary in time and space, which has helped inform forthcoming quantitative analyses of the relationships between exposure to the intervention and the main study outcomes of changes in travel behaviour and physical activity. It has also shown how the intervention has become embedded and entangled within the media discourse surrounding it. This observation has helped shape further analysis of qualitative interview data designed to elicit understanding of the social, as well as the individual, triggers for initiating and maintaining behaviour change in response to the intervention.

In conclusion, by attending to the significance of representations in the media, the knowledge gained from data sources such as the media and qualitative interviews can be used to guide the formulation of hypotheses about how the local population interact with an environmental intervention and the interpretation of quantitative analyses; but it also suggests that evaluation of interventions of this kind should acknowledge and further explore the impact of the social practices illuminated by media analysis rather than merely concentrating on quantitative outcome evaluation.

## Competing interests

The authors declare that they have no competing interests.

## Authors’ contributions

JK and DO conceptualised and designed the study. JK collected, analysed and interpreted the data and drafted the manuscript. DO and SC assisted in data interpretation and revised the manuscript. All authors read and approved the final manuscript.

## Pre-publication history

The pre-publication history for this paper can be accessed here:

http://www.biomedcentral.com/1471-2458/14/482/prepub
